# Developing and Evaluating Newsletters for Parent Engagement in Sustainability via Active Garden Education (SAGE)

**DOI:** 10.3390/ijerph19084617

**Published:** 2022-04-12

**Authors:** Vinny Vi, Bin C. Suh, Elizabeth Lorenzo, Sarah Martinelli, Anel Arriola, Rebecca E. Lee

**Affiliations:** 1 Edson College of Nursing and Health Innovation, Arizona State University, 550 N. 3rd St., Phoenix, AZ 85004, USA; vinnyvi@asu.edu (V.V.); binna.suh@gmail.com (B.C.S.); 2 School of Nursing, University of Texas Medical Branch, 301 University Blvd, Galveston, TX 77555, USA; ellorenz@utmb.edu; 3 College of Health Solutions, Arizona State University, 550 N. 3rd St., Phoenix, AZ 85004, USA; sarah.martinelli@asu.edu; 4 Storytelling Institute, South Mountain Community College, 7050 S. 24th St., Phoenix, AZ 85042, USA; anel.arriola@gmail.com; 5 City of Phoenix Office of Arts and Culture, 200 W. Washington St., 10th Floor, Phoenix, AZ 85003, USA; 6 Center for Health Promotion and Disease Prevention, Edson College of Nursing and Health Innovation, Arizona State University, 550 N. 3rd St., Phoenix, AZ 85004, USA

**Keywords:** childhood obesity, Hispanic, newsletters, parent engagement, healthy lifestyle, early care education

## Abstract

Physical activity and nutrition preschool programming must involve parents in positive long-term healthy habits. This paper describes parent outreach in the Sustainability via Active Garden Education (SAGE) study. Newsletters were sent home with children to promote family opportunities to increase physical activity and fruit and vegetable intake. The content was generated via a community advisory board participatory process. Messages linked SAGE curriculum topics with home and community activities. Parents rated frequency of receipt, helpfulness, satisfaction, and use of content. Most participants were Hispanic (>78%) and women (>95%). Most reported receiving newsletters; nearly all reported that they were helpful. Favorite newsletter components included recipes, pictures of their children and seasonal produce spotlights. Most reported doing physical activities from the newsletters (51.9%). Few reported doing featured physical activity (8.9%) and fruit and vegetable (12.7%) community activities. Newsletter outreach methods are a simple strategy to add value to preschool-based interventions promoting healthy families.

## 1. Introduction

Childhood obesity, defined as a body mass index (BMI) at or above the 95th percentile for height and weight [1], increases the likelihood of developing obesity and chronic diseases in adulthood such as type 2 diabetes and heart disease [2]. Nationally, 13.9% of Hispanic preschool children ages 2–5 are obese [3]—4 times more prevalent than non-Hispanic whites [4]. At the same time, 15% of Hispanic children with parents born in the United States and 17–23% of those with immigrant parents do not participate in any vigorous activity, compared to 9% of non-Hispanic whites [5]. Early care and education (ECE) sites are key locations for interventions that aim to prevent childhood obesity [6], particularly for those participating in nationally sponsored meal and physical activity programming [7].

In addition to ECE influences on a healthy diet and physical activity, parent and family cultural beliefs, access to fresh food, and cooking are also important [8,9], making it key to engage parents and family members alongside ECE-based efforts. Parents may inadvertently encourage overeating to prevent hunger, indulge or reward children with less healthy foods (e.g., candy, sugar-sweetened beverages), perceive their parenting efforts to promote healthy eating as unsuccessful, or face other barriers to healthy eating, such as schedules and lack of access to healthy foods [8,9]. Parenting practices, such as role modeling healthy lifestyles and involving children in family meals and activity planning, can improve child-healthy habits [10]. In addition, parents directly influence the availability of fruits and vegetables at home [11], provide support for children’s physical activity, and discourage sedentary time, depending on the cost of organized physical activity and transportation [9]. Language barriers in Hispanic families can make it harder to find and use physical activity resources outside of the neighborhood [5]. Parent involvement has a salient role in interventions that aim to promote healthy lifestyle habits in preschool children [10].

Engaging parents in school-based interventions that aim to increase healthy eating habits and physical activity generally improves outcomes [11,12,13,14]. Strategies used to engage parents in past interventions include newsletters, phone calls, and the use of workbooks [14]. Nutrition programs centered around the family for children ages 12 and younger increased intake of fruit and vegetables and decreased fat intake [13]. These programs provided simple information and messages to parents about nutrition and followed up with them. Studies with preschoolers and kindergarteners [12,14] showed consistent findings that providing parents with information on increasing fruit and vegetable intake contributed to favorable outcomes in children’s dietary habits and health. These studies have focused on White samples, have not included community-engaged approaches, have not evaluated parents of preschoolers’ perceptions of these materials, and have not been linked to hands-on ECE garden-based learning.

In the Sustainability via Active Garden Education (SAGE) study, a curriculum with a focus on gardening and the use of games and songs was created to teach preschoolers residing in lower-income areas, serving predominantly Hispanic or Latino families about healthy eating and promoting physical activity [15]. Parents received weekly newsletters during the intervention which contained information about activities and resources relating to fruit and vegetable consumption and physical activity at home. The central concerns of this manuscript are to (1) describe the development of the SAGE parent newsletters, (2) summarize the key results of a descriptive analysis of the contents of the weekly newsletters, and (3) examine parents’ perceptions of the newsletters. Findings can provide insight into the effectiveness and process of developing newsletters as parent outreach materials to inform future childhood obesity interventions involving parent engagement in Hispanic or Latino communities in the US.

## 2. Methods

### 2.1. Overview of SAGE

The Sustainability via Active Garden Education (SAGE) study aimed to increase fruit and vegetable consumption and physical activity in preschool children using a curriculum centered around gardening. The curriculum involved interactive activities in the garden and classroom, such as songs, games, fruit, and vegetable tastings, and growing fruits and vegetables. The lessons also discussed healthy eating and its relationship to growth. There were 12 sessions, each one lasted for approximately an hour, and the curriculum met the physical activity and nutrition standards for this age group. The study was conducted at early care and education centers in Phoenix, Arizona and centers were recruited from areas in which the population is more than 30% Hispanic/Latino according to the census [15]. The early care and education centers were randomly assigned to participate in the SAGE intervention or a safety curriculum as the control for comparison, which covered a variety of topics, including swim, fire, bicycle, pedestrian, and stranger safety. Gardens were installed at participating centers before the intervention began. Children were eligible to participate in the study if they were 3–5 years old. Parents were given surveys to determine changes in physical activity, nutrition, and the home environment.

### 2.2. Participants

Participants were mothers of children enrolled in SAGE ECE sites who agreed to answer questions in a post-intervention survey about the newsletters.

### 2.3. Newsletter Development

Newsletters were developed and adapted for this study in collaboration with the SAGE community advisory board (CAB) which consisted of community leaders involved in early childhood education, health and local government, parents, and stakeholders relevant to the Hispanic community. Two nominal group technique (NGT) groups were implemented with the CAB to develop culturally-appropriate messages in the newsletter to promote fruit and vegetable consumption and physical activity participation at home. Such a method is to systematically generate and prioritize topic-specific ideas within a group setting that promotes active participation from all group members [16,17,18,19]. Following a scripted protocol, CAB members were asked to independently generate ideas to increase fruit and vegetable consumption and physical activity participation of children outside the early care and education environment. The members were given ten minutes to brainstorm short messages on each topic, which were then presented one at a time by each CAB member until all ideas were shared. Each message was discussed, refined, and compiled on a master list. The members then rank-ordered their top five messages independently, which were scored according to rankings.

The CAB (*N* = 7) participating in the NGT included four white non-Hispanic/Latino, two white Hispanic/Latino, and one black non-Hispanic/Latino member, including six females and one male. A total of 84 messages were generated, including 36 to promote fruit and vegetable consumption and 48 to increase physical activity participation. The five most highly ranked messages for each topic are presented in Table 1.

Based on the CAB initial feedback, 12 newsletters were created to reinforce curriculum messages from the week as a parent-friendly, home accompaniment to classroom-based learning. The content was developed to include the top-ranked CAB message choices. Web searches were conducted to compile lists of seasonal, locally procurable fruits and vegetables, locations of stores selling fruits and vegetables in bulk or at discounted prices, places to safely do physical activity, and family-friendly, community physical activity events. A bank of easy, healthful recipes was developed that relied on seasonably available, locally procurable fruits and vegetables commonly consumed in Hispanic homes. Lists of the other various items were developed for easy selection and insertion into the newsletter depending on the time of the school year the newsletter was to be distributed. Each newsletter included an activity for children, such as a connect-the-dots or coloring image. All newsletter items were related to fruits and vegetables, physical activity, and the content topic that was featured in the curriculum that week. For example, during the same week when the curriculum discussed hydration and the importance of consuming water over other beverages, the newsletter presented a maze where children and parents could trace the path to help a child find water at the exit of the maze. Another newsletter was developed to include information on hunger and fullness to match lessons in the classroom about understanding how to listen to these sensations. The newsletter activity showed three tummy dolls to color that matched classroom posters, each with the tummy portion of the doll colored in black ink to match a hungry tummy, a “just right” tummy or a too-full tummy. The activity was designed to stimulate conversation between children and parents about hunger and fullness, following the classroom discussion [20].

Newsletters were translated into Spanish. Three native Spanish-speaking parents reviewed newsletter samples in Spanish to ensure they were relevant and accessible. Newsletters were given to teachers during the intervention, who distributed them to parents weekly during child pick-up time [15]. See Supplementary File S1 for samples of newsletters.

### 2.4. Measures

Parents completed paper and pencil surveys prior to intervention programming at Time 1 (T1) baseline and also again after programming at post-test Time 2 (T2). Parents reported their perceptions of the helpfulness of the newsletters on a 5-point Likert scale including answer options “Extremely helpful”, “Very helpful”, “Somewhat helpful”, “Slightly helpful”, or “Not at all helpful”, and the frequency newsletters were received, (once a week, once every other week, once a month, or did not receive the newsletter). Parents were also asked to select their favorite component of the newsletters and in which activities from the newsletter they participated.

### 2.5. Analysis

Components of the curriculum in the newsletters were categorized to describe the content. The PI (Lee), the lead author (Vi) and one co-author (Suh) discussed potential categorization schemes focusing on promoting physical activity and healthy eating at home. A priori specific *elements* had been included in newsletters based on the CAB’s input described above. The lead author and one co-author confirmed the presence of the elements, created definitions, extracted examples and counted the frequency of their appearance. Frequencies of elements that had been included in the newsletters were defined and categorized with examples. Messaging in the newsletters was derived from topics of the SAGE curriculum itself. Message *topics* were confirmed, defined, coded with examples and counted. Parents reported frequency of receipt and satisfaction, as well as favorite aspects of the newsletters, which were evaluated using frequency distributions. Bivariable analyses were conducted to determine whether demographic variables were associated with reporting receiving a newsletter and which elements were reported as the most helpful.

## 3. Results

Table 2 shows the demographic information of participants across all three cohorts in the SAGE study who completed all measures necessary for this analysis; 72.2% of the parents and 74.6% of the children were Hispanic. The parents who participated were mainly women, comprising 95% of the participants. The mean age of the parents was almost 33 years old. Approximately one-third of the parents were married, 20.4% lived together, and 56% had a high school diploma/GED or less. The sex of children who participated in SAGE was 46.3% male and 53.8% female.

Table 3 summarizes the definitions, examples, and frequency of elements of the newsletters (*N* = 12) distributed to parents. The item that appeared most frequently was the list of fruits and vegetables in season (appeared 13 times), followed by recipes (appeared 12 times). The next frequently seen items were physical activity and fruit and vegetable related activities for parents to do with their children (10 times each). These items appeared equally throughout the newsletters, based on the development of the newsletters described above.

Table 4 describes the SAGE curriculum topics mentioned in the newsletters. This included the curriculum topic, definition, examples, and frequencies. These served to inform parents about what their children learned during the SAGE sessions for that week. The newsletters included eight curriculum topics throughout the SAGE intervention. Information about the plant life-cycle appeared most frequently.

Parents reported how often they received newsletters and how helpful they perceived the newsletters to be. Of the 122 parents who completed the survey, one-third (35%) reported never receiving the newsletters. Approximately 29% (*N* = 35) of parents reported receiving the newsletters weekly, 10% (*N* = 12) reported receiving the newsletters every other week, and 26% (*N* = 32) reported receiving them once a month. We conducted bivariable analyses by age, marital status, Hispanic ethnicity, and educational attainment. Reporting receiving newsletters was associated with participant educational attainment. Those with some college or more stated that they had received newsletters less (52%) compared to those with lower educational attainment (high school diploma or equivalent, or less), of whom 76%, reported receiving newsletters, *X*^2^ = 10.716, *p* = 0.013. No other demographic variables were associated with reporting receiving a newsletter. Of those who reported receiving newsletters (*N* = 79), all but one parent reported them to be helpful, with most perceiving them as very helpful (46.9%, *N* = 37), or extremely helpful (16%, *N* = 12).

Table 5 summarizes the responses of participants who answered the parent surveys and who reported receiving at least one newsletter over the course of the SAGE study. Table 5 shows by rank order the parents’ favorite aspects of the newsletters and their reported participation in the activities suggested in the newsletters. Most parents answered that their favorite part of the newsletters was the food recipes (59%) followed by pictures of their children (55.2%), and a list of fruits and vegetables in season (51.3%). Hispanic parents reported that their favorite part of the newsletters was the pictures of children 61% of the time, compared to those who were not Hispanic, mentioning pictures of their children as their favorite part 29% of the time, *X*^2^ = 4.945, *p* = 0.026. No other significant differences were noted. Regarding participation in the activities in the newsletters, some parents reported participating in physical activities (51.9%) and fruit and vegetable activities (36.7%) at home. Participation in community activities was low, with 8.9% of parents participating in community physical activities and 12.7% of parents participating in community activities related to fruits and vegetables.

## 4. Discussion

Overall, the most frequent elements that appeared throughout the newsletters included lists of fruits and vegetables in season, recipes, and physical activity/fruit and vegetable activities to try at home, reflecting the aims of the parent study and confirming the content validity of the newsletters. The favorite element of the newsletters for most parents was the food recipes, and the third most favorite was the list of fruits and vegetables in season, reflecting the two most frequently appearing items. The second most favorite newsletter element was the pictures of the children.

Reported participation in the community events related to physical activity or fruit and vegetable consumption was lower compared to events completed at home, including the park locater and grocery sales. Most of the respondents reported participating in physical activity ideas or suggestions from the newsletters either at home or in the community, lower than measured population levels of physical activity. More of this was reported participation in home-based activities, while few reported participating in community physical activities (<9%). These rates are consistent with the overall low rates of physical activity in US Hispanic children, which ranged from 15–23% who did not participate in vigorous physical activity [5].

About half of all the respondents reported participating in fruit or vegetable activities either at home or in the community. Activities at home (e.g., trying a suggested recipe) were more commonly endorsed than community events (<13%). In addition, on average, US Hispanic adults do not consume the recommended amount of fruits and vegetables, which may have affected participation rates in the suggested fruit and vegetable activities. In comparison to the recommended amount of 1.5–2 cups of fruit and 2–3 cups of vegetables daily, US Hispanic adults on average consume 0.78 cups of fruit and 1.33 cups of vegetables daily [21].

Both home-based participation in physical activity and fruit and vegetable activities were more popular among parents compared with community-based participation. This may suggest that parents prefer activities that can be easily done at home, or there may be other factors limiting their ability to participate in community events. One study interviewed Hispanic parents who reported time or scheduling as factors that limited their ability to eat healthfully or participate in physical activities [9]. Furthermore, socioeconomic factors have long been noted as a barrier to regular physical activity for low-income families, including lack of access to safe places to play in the community or time constraints of working multiple jobs [22]. Some parents in previous interviews also reported not having the knowledge to prepare vegetables if their children disliked them [9]. The recipes offered in the newsletters were the parents’ favorite elements and involved clear instructions on how to prepare simple dishes with vegetables or fruits. The use of easy to follow, culturally-tailored recipes may potentially help mitigate a lack of knowledge about vegetable preparation and increase consumption.

Interestingly, the fruit and vegetable related aspects of the newsletters were favored compared to the physical activities; however, parents reported participating in more home-based physical activities compared to fruit and vegetable activities. This may be explained by the time and knowledge needed to prepare nutritious recipes [9], even easy ones, whereas physical activities suggested in the newsletter did not take additional planning. Perhaps parents may choose ease of participation over personal preference, necessitating future research.

The majority of the parents participating in the SAGE study perceived the newsletters to be helpful. The newsletters provided recipes and activities to promote fruit and vegetable consumption and physical activity while informing parents of what was learned in the SAGE curriculum. Newsletters are a simple and affordable way to reinforce what was learned in the school setting. This presents opportunities for parents to engage with their children and become involved with their nutrition and physical activity, which promotes retention of information and transfers learning from the school setting to the home environment for value-added intervention impact.

A limitation was not all parents reported that they had received the newsletters. Out of 122 participants who were recruited for this study, 79 participants reported that they had received the newsletters. Anecdotal notes from intervention implementers suggested that sometimes newsletters were not distributed by busy teachers, and it is unclear whether parents received them from the ECE center. Other limitations include missing responses for some questions and parents not returning surveys, which could affect the results. Finally, surveys did not include specific questions delineating in which specific fruit and vegetable or physical activities parents participated.

This study has several strengths that could offset the limitations. The method of counting frequencies to conduct the descriptive analysis of the newsletters is objective and rigorous [23]. The coding of the categories in the newsletters limits bias. This is because the author who participated in the analysis was not involved in the implementation phase of the SAGE study. In addition, the survey responses provided a clear way to quantify and determine the level of satisfaction parents had with the newsletters and identify which aspects of the newsletters they preferred the most. The SAGE study was conducted at multiple ECE centers in order to increase access to the population and the replicability of the study findings. It is also noteworthy that the newsletters were developed based on narratives of people in the communities where the study took place. The newsletters were tailored to the food availability in the area through the reminders to check local sales and applicable discounts. There were local opportunities offered for physical activity, as well. Furthermore, staff took pictures of the children during interventions and shared the pictures with their parents in the weekly newsletters. This makes the newsletters more personal and tailored to the families who participated in the study. The newsletters were translated into Spanish for the Hispanic/Latino parents who preferred the Spanish language.

Future research should include conducting detailed interviews with parents to gain more information about their perception of the newsletters and which specific activities they participated in most. The parents’ knowledge of healthy eating and physical activity could be assessed to determine what type of information could be prioritized in the newsletters. It is recommended that newsletters be used in future interventions to address childhood obesity to promote physical activity and the consumption of fruits and vegetables. Future research should investigate whether program uptake and health outcomes are associated with improved parent engagement. Newsletters are potentially more accessible than electronic means of contact. Future research should investigate adding additional electronic elements, such as text messages. Newsletters are a simple and inexpensive way to reach parents in underserved communities and transfer knowledge and information from the classroom to the home environment.

## 5. Conclusions

The newsletters were mainly perceived as helpful by parents of the children who participated in the SAGE intervention. As the most favorite elements of the newsletters were the recipes, the list of in-season fruit and vegetables, and pictures of children, these elements can be further developed in future childhood obesity interventions utilizing newsletters. Culturally and locally tailoring these components of the newsletters was helpful, as personalization can encourage parent engagement and make the activities easier to participate in. Translating newsletters into Spanish and including photos of children made them more personalized, as well.

Overall, greater participation was reported for home-based versus community-based activities. Looking at the home-based activities, there was more participation in physical activities versus fruit and vegetable activities. The higher participation in home-based activities suggests that future newsletters can place emphasis on at-home activities to encourage physical activity and fruit and vegetable intake. Recommended activities can be simple and easy to complete to help alleviate possible barriers and time constraints that limit parents’ ability to participate in community activities. Future studies are needed to investigate how receiving newsletters from a parent’s school can influence, initiate and increase participation, using robust measures of individual and community physical activity and fruit and vegetable activities.

Using newsletters in early childhood obesity interventions is an effective way to engage parents in their child’s nutrition and physical activity. Newsletters can encourage healthy habits, reinforce information about healthy eating and physical activity, and increase awareness of local opportunities. Creating positive changes in nutrition and physical activity habits at home early on can have a long-term impact on mitigating childhood obesity in the Hispanic population.

## Figures and Tables

**Table 1:** Summary of the most highly ranked messages generated from Community Advisory Board meetings for fruit and vegetable consumption or physical activity home participation.

Topic
**Fruit and Vegetable**
Kids help shop, purchase, wash & prepare fruits and veggies
Plant a garden and grow fruits and veggies in order to get children interested in eating them
Choose a “fruit of the [month, week, day]” and learn different ways to prepare it
Parents should model eating fruits and veggies and discuss positively
Offer fruits and veggies at snack time instead of other foods or choose fruit as a bedtime snack
**Physical Activity**
Dance together
Children are happiest when they have time to be active/children are happy when they are moving
Go on a nature walk and collect items
Visit a local park and play tennis, dodge ball, etc.
Play catch with your children
Gardening at home is fun and rewarding
Get possible places where they have exercise activities (City of Phoenix, Department of Parks and Recreation, YMCA)

**Table 2:** Sample characteristics of SAGE participants (*N* = 122).

Category	*N* (%)
**Parent**	
Sex	
Male	6 (5.0)
Female	113 (95.0)
Age in years (M/SD)	32.72 (7.85)
Hispanic	88 (72.2)
Marital status	
Married	38 (35.2)
Living together	22 (20.4)
Separated, divorced, widowed	18 (16.7)
Single (never married)	30 (27.8)
Education	
High school/GED or less	61 (56.0)
Some College or higher	48 (44.0)
**Child**	
Sex	
Male	55 (46.3)
Female	64 (53.8)
Age in months (M/SD)	52.20 (5.03)
Hispanic	88 (74.6)

Note. Numbers may not add up to 122 due to missing responses.

**Table 3:** Definitions, Examples, and Frequency of Newsletter Elements.

Element	Definition	Examples	Frequency in Newsletters (Counts)
Fruits/vegetables in season	List of fruits and vegetables in season	Buy Fruits and Vegetables in Season Cilantro, radishes, oranges, beans, etc.	13
Recipe	Recipe with ingredients and directions	Recipe of the Week Greens with Radishes and Snap Peas	12
Physical activity at home	Physical activity suggestion to participate in with child	Ideas for Home! Create daily physical activity calendar, jumping jacks, 1 min of dancing, play tag, etc.	10
Fruit/vegetable activity at home	Activity to encourage fruit and vegetable consumption	Ideas for Home! Put out a variety of fruits and vegetables, let child prepare own kebab, ask child to pick out a fruit or vegetable they haven’t tasted at grocery store and try it together	10
Grocery sale check	Reminder to check weekly ad for sales	Did you know? Grocery stores have 3-day sales, be sure to check weekly ad to see produce weekend sales	9
Grocery purchase	Information about purchasing produce for low price	Did you know? You can buy up to 60 lbs. of fresh produce	4
How to start garden at home	Link to start own garden at home	Ideas for Home! Preschoolers love to learn about where fruits and vegetables come from. For ideas on how to start your own garden visit	1

Note: A total of 12 newsletters were evaluated.

**Table 4:** Summary of SAGE curriculum topics included in newsletters.

Curriculum Topic	Definition	Example	Frequency in Newsletters (Counts)
Plant life cycle	Growth stages of plants from seed to fruit bearing and requirements for plant growth	Unit 1: “learned how fruits and vegetables come from seeds” “life-cycle of plants, from seed to sprout, seedling, mature plan, and finally, producing fruit” Unit 2: “played a game called “Plant Race.” “helps children learn how seeds need soil, sunlight, and water to grow healthy and strong” Unit 5: “reviewed the plant growth process”	3
Variety	Eating a variety of fruits and vegetables every day is needed for growth, strength, and health	Unit 1: “how children need to eat many fruits and vegetables to grow strong and healthy” Unit 8: “reviewed how they need to eat a variety of fruits and vegetables every day” “how it is important to eat a variety of fruit and vegetables to grow and be healthy”	2
Comparison between needs of plants and people	Plants and people have specific needs for good healthy growth and strength	Unit 5: “just as plants need water and nutrients to grow, we need water, fruits, and vegetables to grow healthy and strong” Unit 10: “reviewed how plants need soil, sunlight, and water, and humans need fruits, vegetables, and physical activity to stay health and grow strong”	2
Physical activity	Effects of physical activity and role of gardening	Unit 3: “learned about how physical activity gives the body energy” “talked about how being physically active every day will help keep them healthy and strong” Unit 9: “learning how gardening is a way to be physically active”	2
Effects of food on the body	Food provides nutrients and energy for activity. Different parts of the body benefit from different foods	Unit 6: “how food gives us nutrients” “how different foods are good for different parts of the body” “foods give us energy to function and be active”	1
Hydration	Effect of not drinking enough water	Unit 4: “plants and people feel tired when they don’t drink enough water”	1
Identifying fullness and hunger	Bodies convey fullness and hunger, important to notice when to eat or stop	Unit 7: “our bodies let us know when we feel hungry or full” “it is important to listen to our bodies to know when we need to eat and stop eating”	1
Harvesting/ripeness	Characteristics that indicate progression towards harvesting and ripeness	Unit 11: “learned about harvesting” “learned how to look at changes in color and size, how “ripe” fruits and vegetables smell”	1

Note: The unit number before each example indicates the number of the curriculum unit in which the example was found. Website with full curriculum information available from the authors.

**Table 5:** Favorite Aspects of Newsletters and Participation (*N* = 79).

What were your favorite parts of the newsletters?	*N* (%)
Food recipes	46 (59.0)
Pictures of my kid(s)	43 (55.2)
The list of fruits and vegetables that are in season	40 (51.3)
Updates on SAGE	32 (41.0)
Songs or games to participate in with your kid(s)	19 (24.4)
Community events	16 (20.5)
Other	4 (5.1)
None	0 (0.0)
**Did you participate in any of the following suggested activities from the newsletters or text messages?**	***N* (%)**
Physical activities within my own home	41 (51.9)
Fruit and vegetable related activities within my own home	29 (36.7)
None	24 (30.4)
Fruit and vegetable related activities within the community	10 (12.7)
Physical activities within the community	7 (8.9)
Other	3 (3.8)

## Data Availability

Data are available from authors.

## Supplementary Materials

The following supporting information can be downloaded at: https://www.mdpi.com/article/10.3390/ijerph19084617/s1, File S1: Sample parent newsletters from the Sustainability via Active Garden Education project.
