# Supplementary material for: Developing and Evaluating Newsletters for Parent Engagement in Sustainability via Active Garden Education (SAGE)
Source: Int J Environ Res Public Health. 2022 Apr 12;19(8):4617. doi: 10.3390/ijerph19084617 (PMC9025365; doi:10.3390/ijerph19084617)

File S1. Sample parent newsletters from the Sustainability via Active Garden Education project.

SAGE
Sustainability via Active Garden Education

This week in SAGE

The first session of SAGE began this week. The children learned how fruits and vegetables come from seeds. They learned about the life-cycle of plants, from seed to sprout, seedling, mature plant, and finally, producing fruit. We talked about how children need to eat many fruits and vegetables to grow strong and healthy. Ask your child to sing the “Seedling Song” with you!

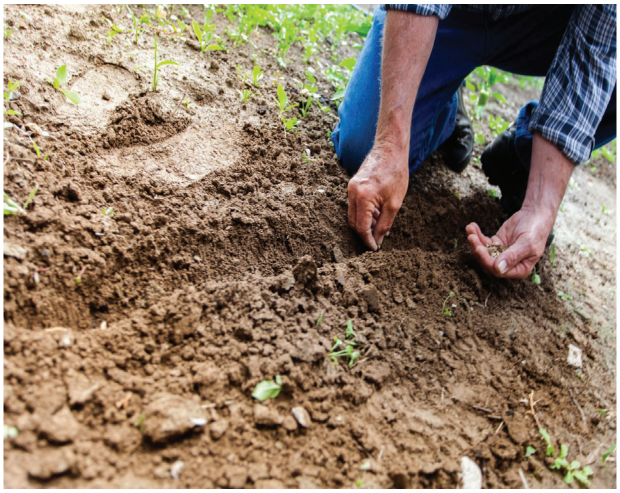

Garden Update

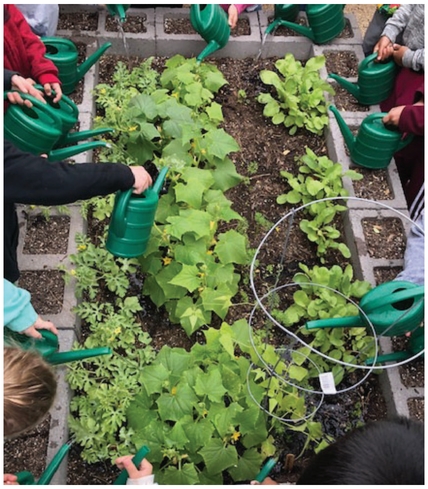

We started our SAGE garden this week! We will be growing kohlrabi, spinach, lima beans, and cucumbers.

Ideas for Home!

Activity idea! Create a monthly or weekly calendar with daily physical activities for your child to do! Try 25 jumping jacks or 1 minute of silly dancing!

Gardening at home is fun and rewarding. Preschoolers love to learn about where fruit and vegetables come from. For ideas on how to start your own garden visit https://bit.ly/2Gf5K6U.

Help your child connect the dots on the radish! Let your child color it after.

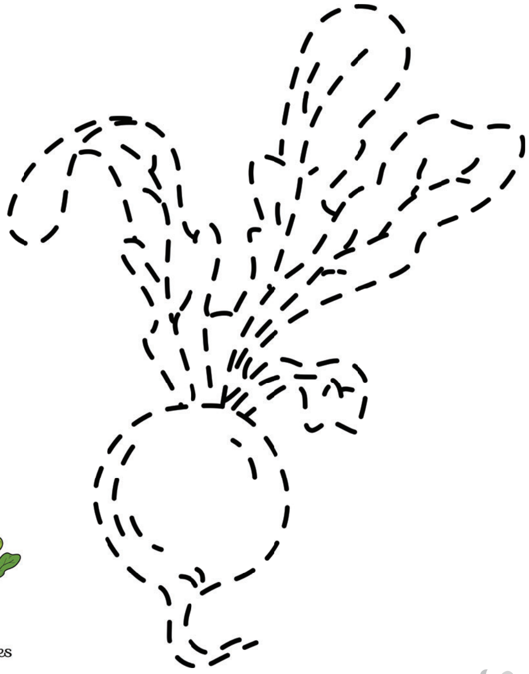

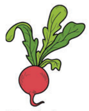

Buy fruits and vegetables in season

- Beets
- Broccoli
- Cabbage
- Cauliflower
- Carrots
- Celery
- Grapefruit
- Lemons
- Oranges
- Spinach
- Strawberries
- Tangerines

Recipe of the Week:

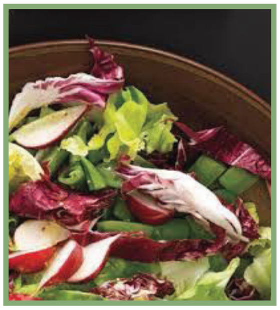

Greens With Radishes and Snap Peas

Ingredients:

3 tablespoons olive oil
1/2 teaspoon sugar
1/2 teaspoon salt & pepper
1 large head lettuce
1 bunch radishes, cut into thin wedges
1/2 pound snap peas

Directions: In a large bowl, whisk together the oil, sugar, salt, and pepper. Add the lettuce, radishes, and snap peas and toss to combine. Ready!

For more events and resources, check out our website at https://sites.google.com/asu.edu/sageasu/home?authuser=0

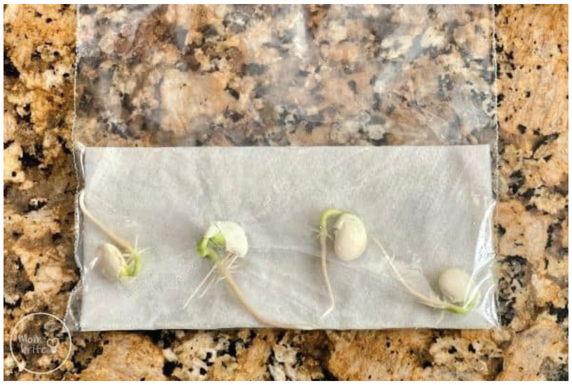

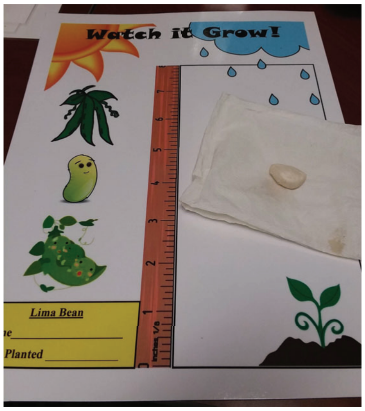

This week children learned how to plant a lima bean using a paper towel. Just like all plants, lima beans need water and sunshine to grow!

Did you know:

Grocery stores have 3-day sales?
Be sure to check your weekly ad to see produce weekend sales.

For some family physical activity visit the website below to find a park near you. https://www.discovertheforest.org. Select "Locate a forest or park," enter your zip code and find your nearest park!

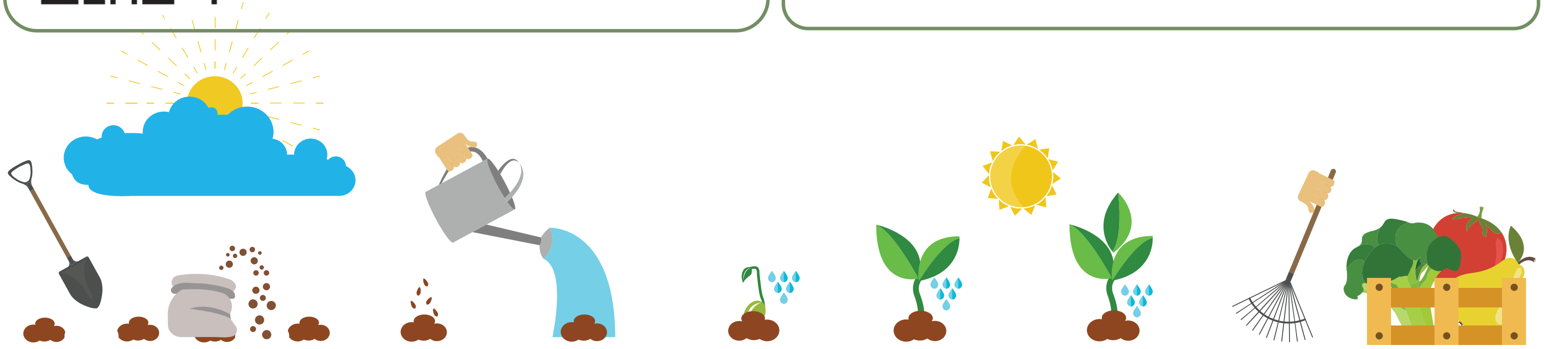

SAGE

Sustainability via Active Garden Education

Esta semana en SAGE

La primera sesión de SAGE empezó esta semana. Los niños aprendieron que las frutas y verduras vienen de las semillas. Aprendieron acerca del ciclo de vida de las plantas, desde la semilla, germinación, plántula, planta adulta, y el fruto. ¡Pídale a su hijo(a) que cante la canción "Seedling Song" con usted!

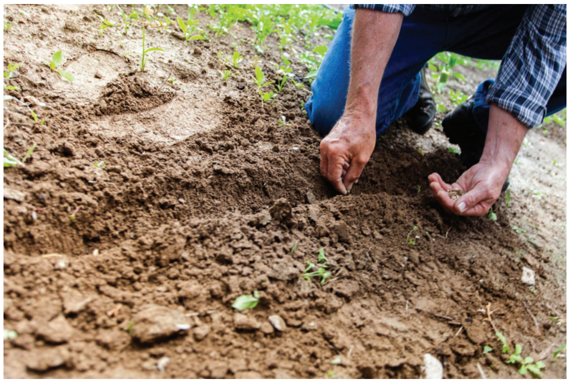

Al día con el jardín

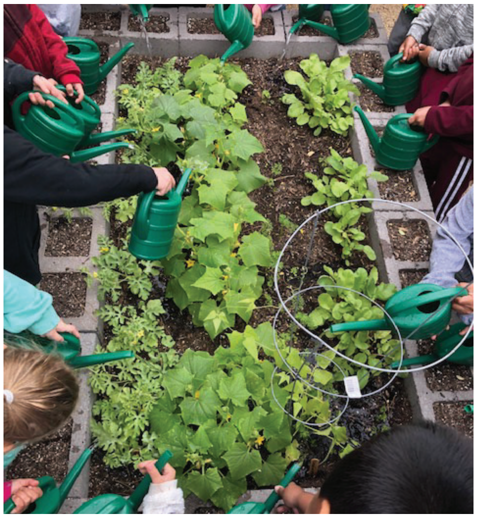

¡Empezamos nuestro jardín de SAGE esta semana! Plantamos semillas de colinabo, espinaca, habas, y pepino.

¡Ideas para el hogar!

¡Idea para una actividad! ¡Haga un calendario semanal o mensual con actividades físicas diarias que pueda hacer su hijo(a)! ¡Intente 25 saltos de tijera o 1 minuto de baile chistoso!

El cuidado de un jardín en el hogar es divertido y gratificante. A los niños de edad prescolar les encanta aprender acerca de donde vienen las frutas y las verduras. Empiece su propio jardín o participe en un jardín comunitario. Para ideas visite https://bit.ly/2E2cCig.

¡Ayude a su hijo(a) a conectar los puntos del rábano! Deje que su niño(a) lo coloree después.

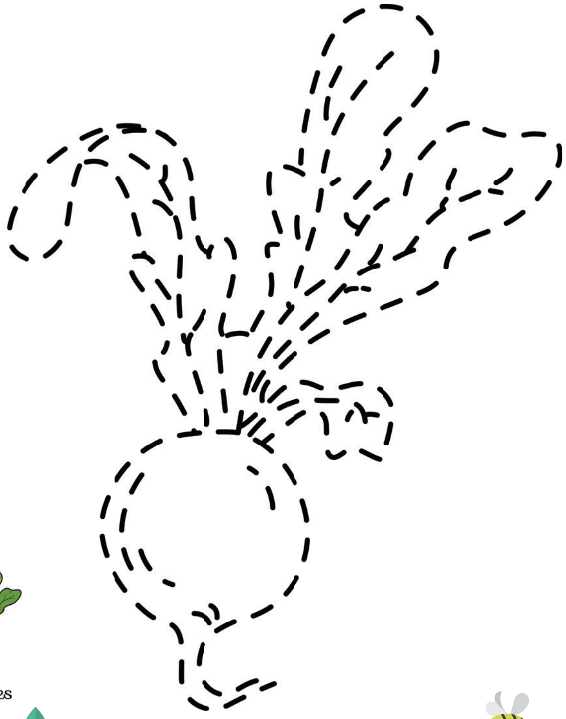

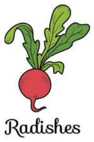

Compre frutas y verduras que estén en temporada

Remolacha	Pomelo
Brócoli	Limón
Repollo	Naranjas
Coliflor	Espinacas
Zanahorias	Fresas
Apio	Mandarinas

Receta de la semana:

Verduras de hoja verde con rábanos y chícharos de vaina

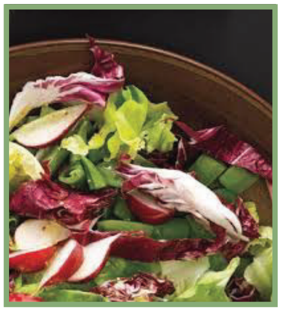

Ingredientes:

3 cucharadas aceite de oliva
1/2 cucharadita de azúcar
1/2 cucharadita de sal y pimienta
1 cogollo de lechuga
1 racimo de rábanos, cortados en rajas delgadas.
1/2 libra chícharos de vaina

Instrucciones: En un plato hondo grande, bata el aceite, azúcar, sal y pimienta. Agregue la lechuga, rábanos, y chícharos de vaina y mezcle para combinar. ¡Listo!

Para más eventos y recursos, visite nuestro sitio web en https://sites.google.com/asu.edu/sageasu/home?authuser=0

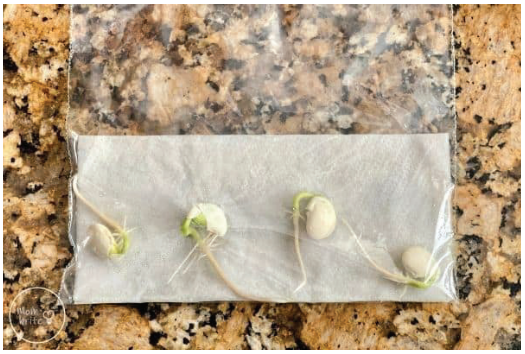

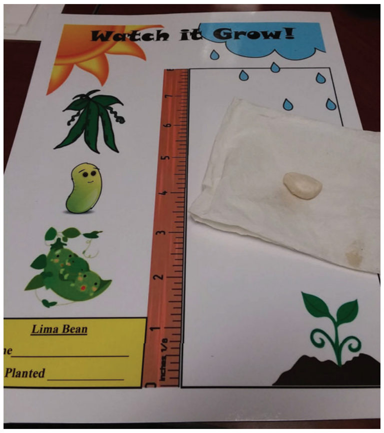

Esta semana los niños aprendieron como plantar un haba usando una toalla de papel. ¡Así como todas las plantas, las habas necesitan agua y sol para crecer!

¿Sabía usted?

Los supermercados tienen ventas de 3 días. Asegúrese de revisar el anuncio semanal para ver las ventas de frutas y verduras del fin de semana.

Para actividad física en familia visite el sitio web abajo para encontrar un parque cerca de usted. http://www.descubreelbosque.org/ Seleccione ¡"Localiza un parque cerca de ti" ponga su código postal y encuentre su parque más cercano!

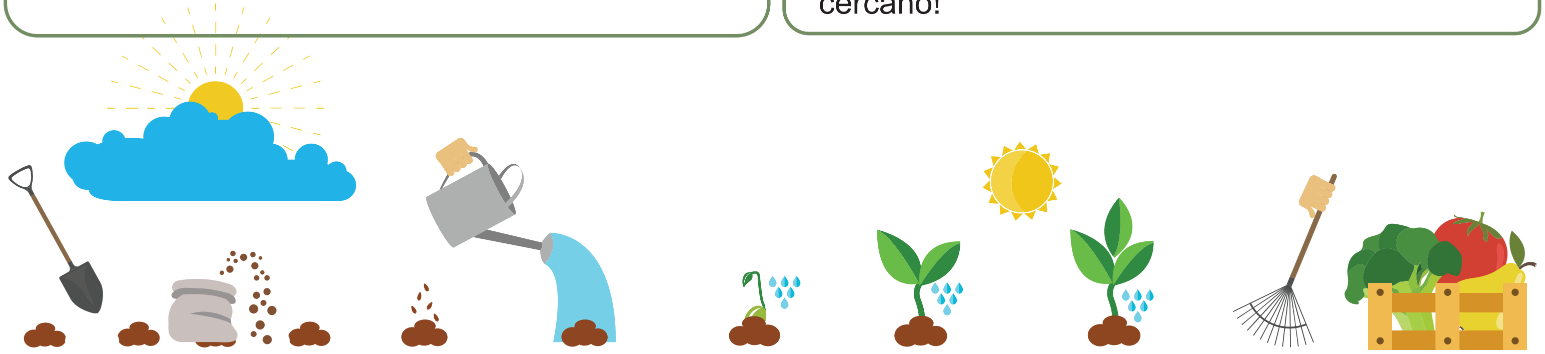

SAGE

Sustainability via Active Garden Education

This week in SAGE

This week children learned about hunger and fullness. Our bodies let us know when we feel hungry or full. It is important to listen to our bodies to know when we need to eat and stop eating. We tasted fruits and vegetables during the session. The children practiced identifying how they feel before the tasting using the Tummy Dolls.

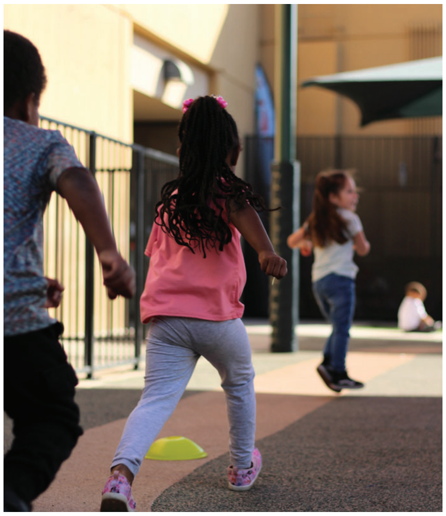

Garden Update

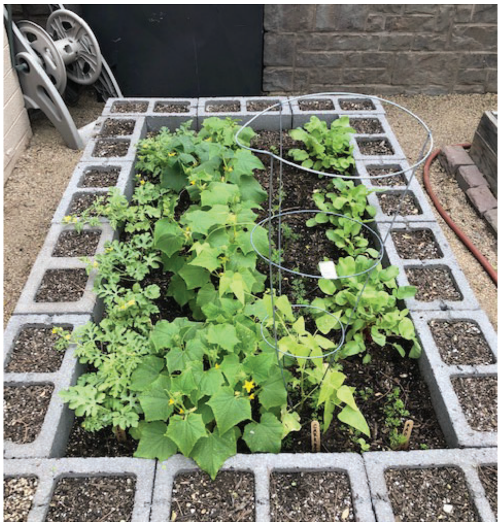

Love and attention not only help us to thrive, but they help our gardens grow as well! Look how big our plants our getting!

Ideas for Home!

Activity idea! Play tag or chase with your child to be active today!

Buy fruits and veggies in season- Try to buy fruits and veggies that are in season! Find out what is in season: http://bit.ly/2qAhOUU.

Help your child identify if he/she is hungry, just right, or too full. Let your child color the tummy dolls.

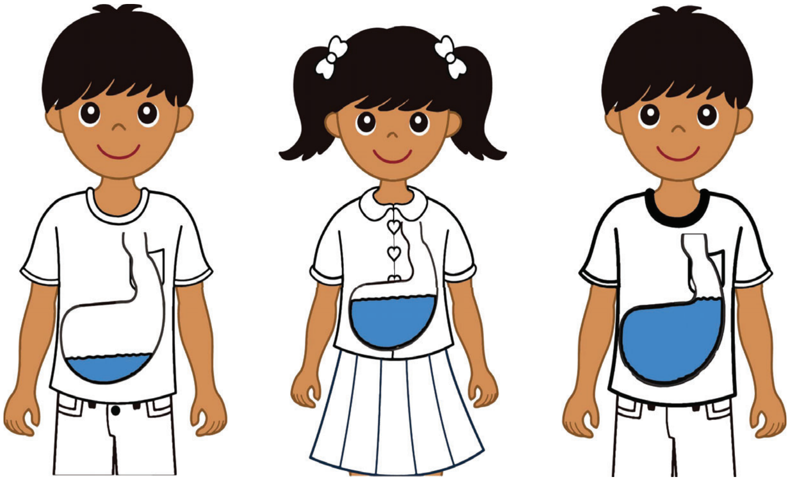

Hungry | Just right | Too full

Buy fruits and vegetables in season

Asparagus	Carrots
Beets	Celery
Dates	Grapefruit
Broccoli	Lemons
Cabbage	Oranges
Cauliflower	Spinach

Recipe of the Week:

Tangerine Butterfly

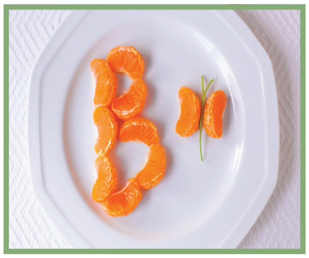

Ingredients:

1 tangerine
1 baby spinach leaf

Directions:

Peel the tangerine and separate the segments. Lay two of the pieces out, round edges together, to form a butterfly. Arrange the rest of the pieces into the letter B.

I found this was easiest to do with 8 pieces. Use a paring knife to carefully cut out the stem of the spinach leaf and then split the widest part of the stem in half.

This forms the butterfly body with antennae. Place the butterfly body between the two tangerine segments. An easy way to turn a tangerine into a cute alphabet lesson!

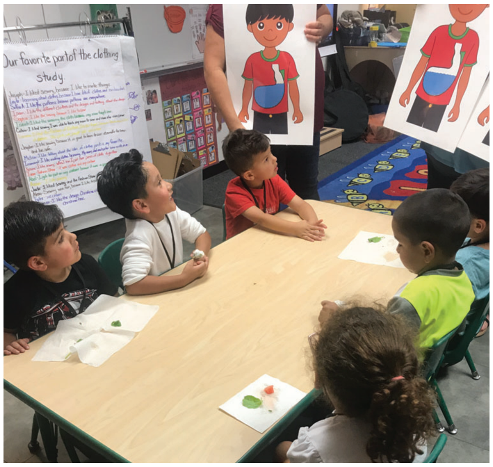

We lead the children through a mindful eating activity and let them try different fruits and vegetables.

Did you know:

You can buy up to 60 lbs of produce for $10! Check the website for a stop near you: http://www.borderlandsfoodbank.org/produce-on-wheels/

For some family physical activity go to the nearby park to walk. Check out the website to locate a park near you with zip code: https://www.phoenix.gov/parks/parks

For more events and resources, check out our website at https://sites.google.com/asu.edu/sageasu/home?authuser=0

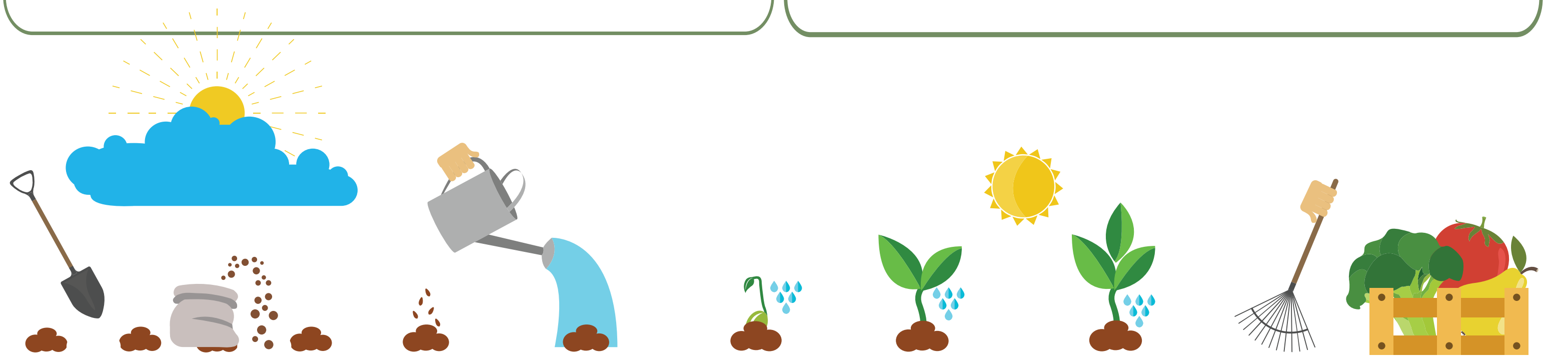

SAGE

Sustainability via Active Garden Education

Esta semana en SAGE

Esta semana los niños(as) aprendieron acerca del hambre y sentirse satisfecho. Nuestros cuerpos nos dejan saber cuando tenemos hambre o cuando nos sentimos llenos. Es importante escuchar a nuestro cuerpo para saber cuando necesitamos comer y cuando dejar de comer. Probamos frutas y verduras durante la sesión. Los niños practicaron identificar como se sienten antes de la degustación usando Tummy Dolls (Imagenes de muñequitos con hambre, satisfecho, y sin hambre).

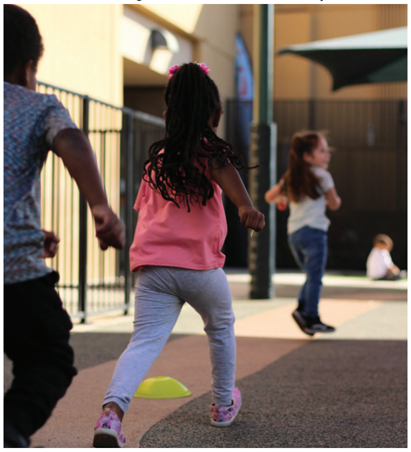

Al día con el jardin

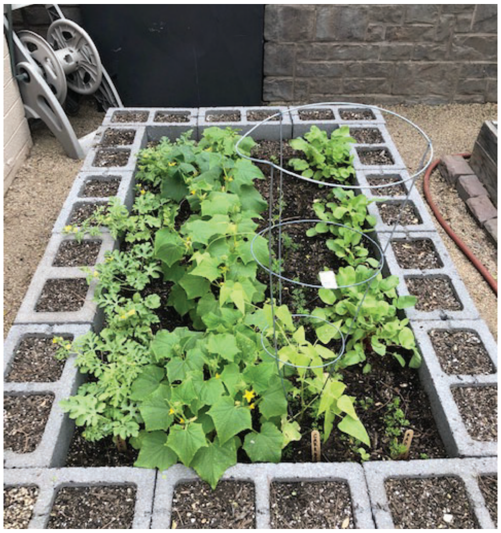

¡El amor y la atención no sólo nos ayudan a prosperar, pero ayudan nuestros jardines a crecer también! ¡Mira que grandes están creciendo nuestras plantas!

¡Ideas para el hogar!

¡Idea para una actividad! ¡Juegue a las traes o a perseguir a su niño(a) para estar activo(a) hoy!

Compre frutas y verduras en temporada. ¡Trate de comprar frutas y verduras en temporada! Para encontrar que está en temporada: http://bit.ly/2qAhOUU.

¡Ayude a su niño(a) a identificar si tiene hambre, esta satisfecho(a), o muy lleno(a)! Deje que su niño(a) coloree los muñecos después.

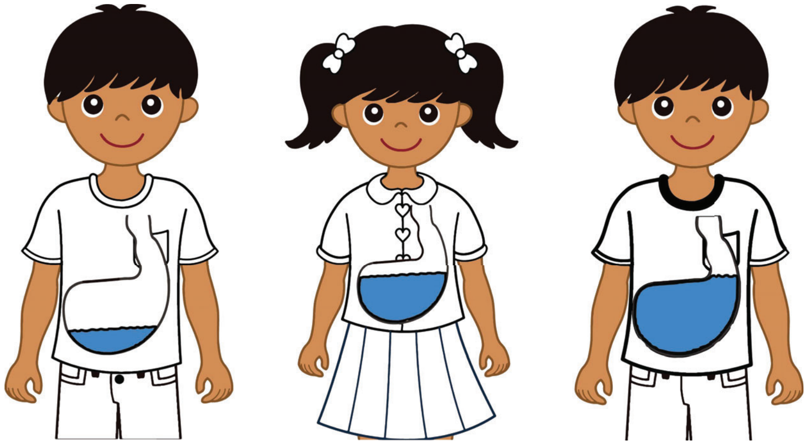

Con hambre | Satisfecho(a) | Lleno(a)

Compre frutas y verduras en temporada

Espárragos	Zanahorias
Remolacha	Apio
Fechas	Pomelo
Brócoli	Limones
Repollo	Naranjas
Coliflor	Espinacas

Receta de la semana:

Mariposa de Mandarina

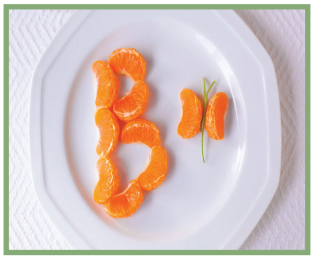

Ingredientes:

1 mandarina

1 hoja de espinaca

Instrucciones:

Pele la mandarina y separe los pedazos. Ponga dos de los pedazos juntos, con las partes redondas tocando, para formar una mariposa. Coloque el resto de los pedazos en forma de la letra B.

Descubrí que esto es más facil de hacer con 8 pedazos. Use un cuchillo de pelar para cortar la raíz de la hoja de espinaca y luego corte la parte más amplia de la raíz a la mitad.

Esto forma el cuerpo de la mariposa con antenas. Coloque el cuerpo de la mariposa entre los dos pedazos de mandarina. ¡Una manera facil de convertir una mandarina en una clase de alfabeto!

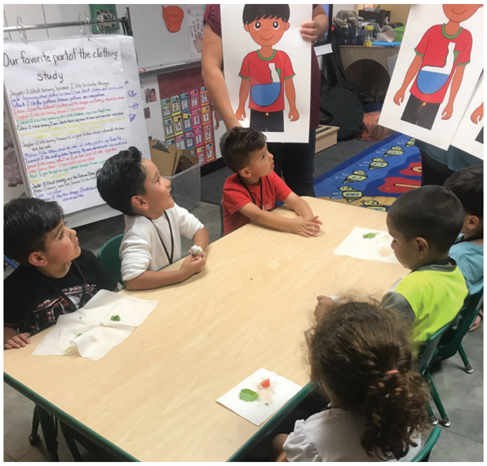

Guiamos a los niños en una actividad sobre alimentos saludables y les dimos a probar diferentes frutas y verduras.

¿Sabía usted?

¡Puede comprar hasta 60 libras de verduras y frutas por $10! Visite el sitio web para una parada cerca de usted: http://www.borderlandsfoodbank.org/produce-on-wheels/

Para actividad física en familia vaya al parque cercano para caminar. Visite el sitio web para encontrar un parque cerca de usted con su codigo postal: https://www.phoenix.gov/parks/parks

Para más eventos y recursos, visite nuestro sitio web en https://sites.google.com/asu.edu/sageasu/home?authuser=0

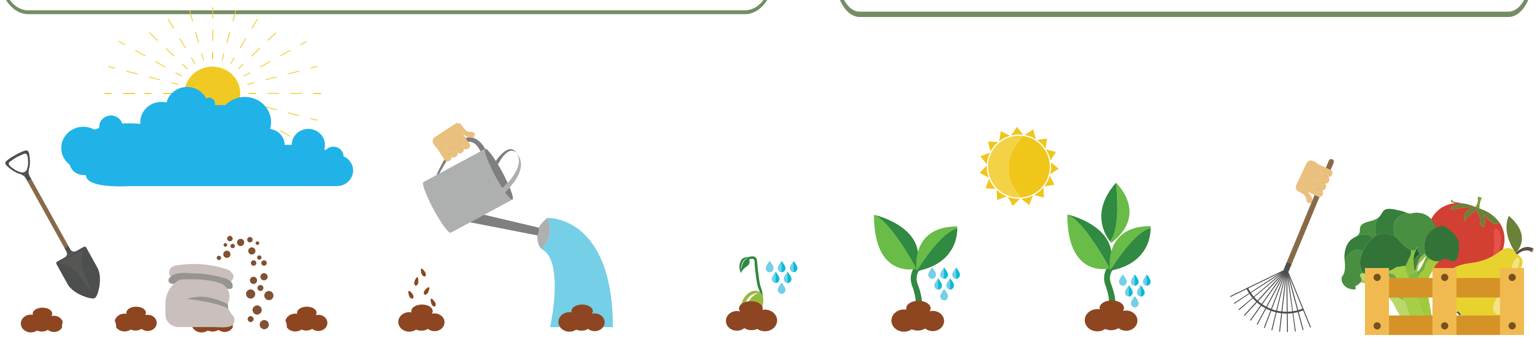

SAGE

Sustainability via Active Garden Education

This week in SAGE

This week we read the book, "The Little Gardener." The children acted out each page of the book. They are learning how gardening is a way to be physically active. We watered the garden and played "Plant Splash Relay" to get some physical exercise. Ask your child to act out ways he or she can be physically active in the garden.

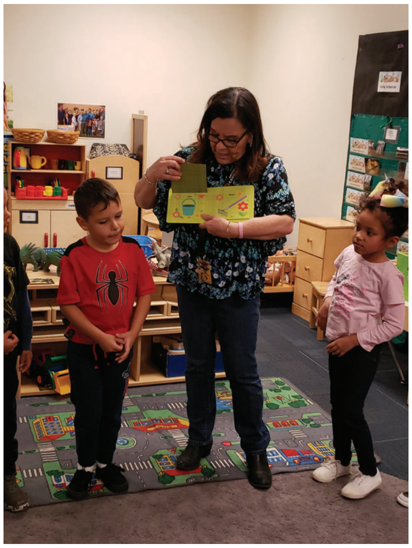

Garden Update

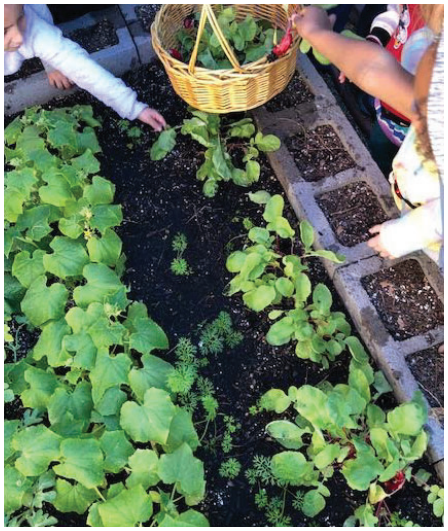

We planted Broccoli and Celery in the garden!

Ideas for Home!

Limit TV, video game, and mobile device use by your preschooler. Encourage your child with active play instead!

Did you know broccoli can lower your risk for some cancers? Prepare as a side dish or add to your favorite recipe.

Help your child connect the dots on the pumpkin! Let your child color it after.

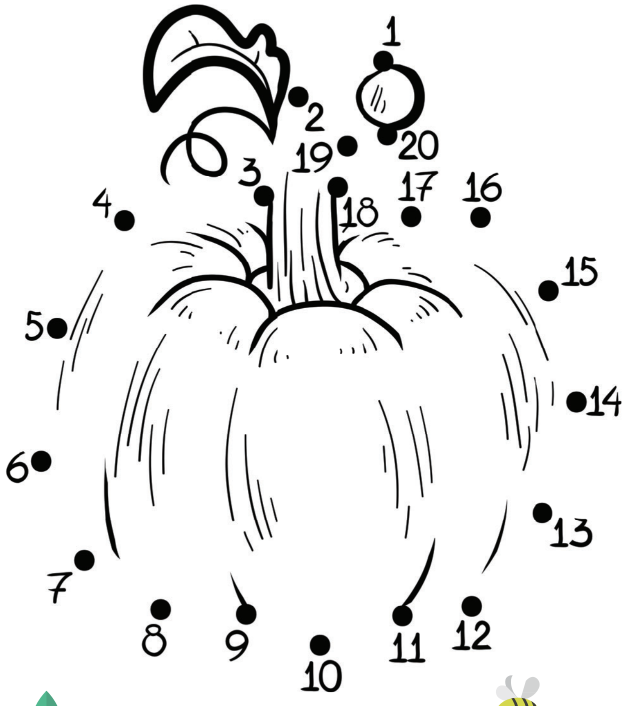

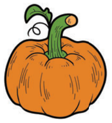

Buy fruits and vegetables in season

Apple
Beets
Dates
Eggplant
Spinach
Tangerines
Tomatoes
Grapefruit
Lemons
Oranges
Celery

Recipe of the Week:

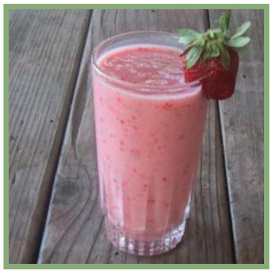

Fresh Grapefruit Juice Smoothie

Ingredients:

1 1/3 cups fresh red grapefruit juice
8 large strawberries
2 medium bananas, sliced
8 oz strawberry-banana yogurt
2 tablespoons honey
1 cup crushed ice

Directions: Place the grapefruit juice, strawberries, bananas, yogurt, honey, and ice into a blender. Cover, and blend until smooth.

For more events and resources, check out our website at https://sites.google.com/asu.edu/sageasu/home?authuser=0

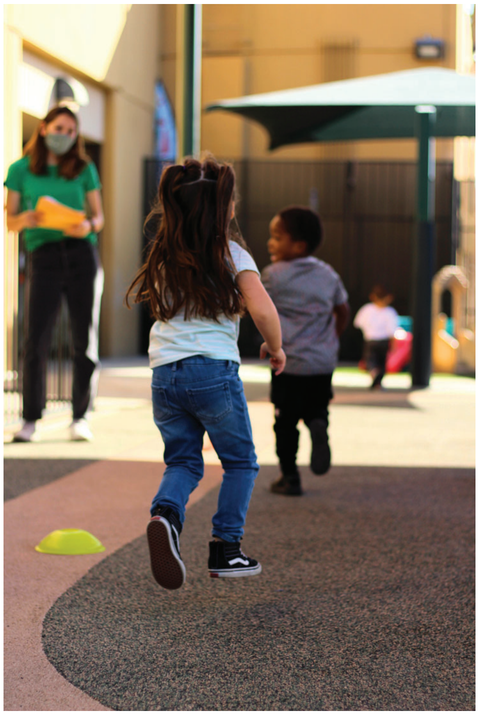

The children are getting physical activity while tending the garden. Gardening can teach communication and observation skills!

Did you know:

Grocery stores have 3-day sales? Be sure to check your weekly ad to learn about produce weekend sales.

For some family physical activity visit the website below to find a park near you. https://www.discovertheforest.org. Select "Locate a forest or park," enter your zip code and find your nearest park!

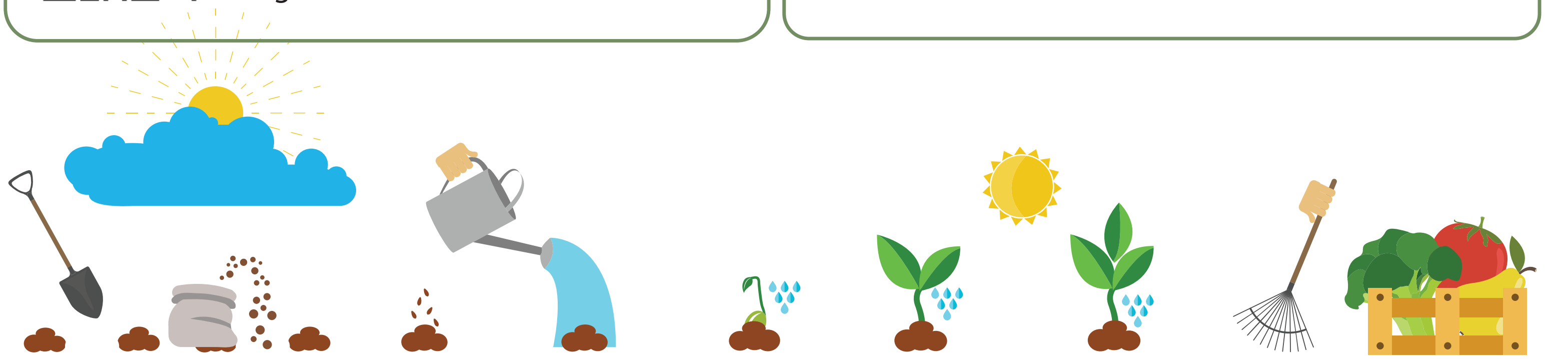

SAGE

Sustainability via Active Garden Education

Esta semana en SAGE

Esta semana leímos el libro "The Little Gardener." Los niños actuaron cada página del libro. Están aprendiendo como la jardinería es una manera de estar activo físicamente. Regamos el jardín y jugamos "Plant Splash Relay" para hacer más actividad física. Pídale a su niño(a) que le enseñe maneras en las cuales puede estar físicamente activo(a) en el jardín.

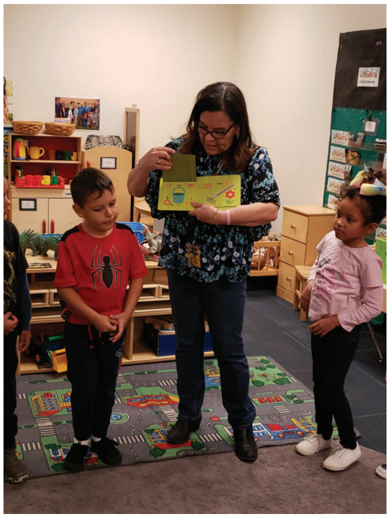

Al día en el jardín

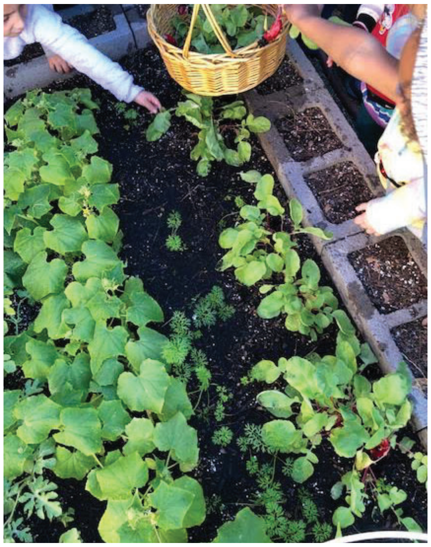

Nosotros plantamos brócoli y apio.

¡Ideas para el hogar!

Limite el uso de la televisión, videojuegos, y aparatos móbiles de su niño(a) de edad prescolar. ¡En vez, anime a su niño(a) con juegos activos!

¿Usted sabía que el brócoli puede disminuir el riesgo de contraer ciertos tipos de cáncer? Preparelo como un aperitivo o agreguelo a su receta favorita.

¡Ayude a su niño(a) a conectar los puntos en la calabaza! Deje que su niño(a) la coloree después.

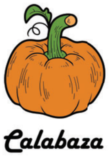

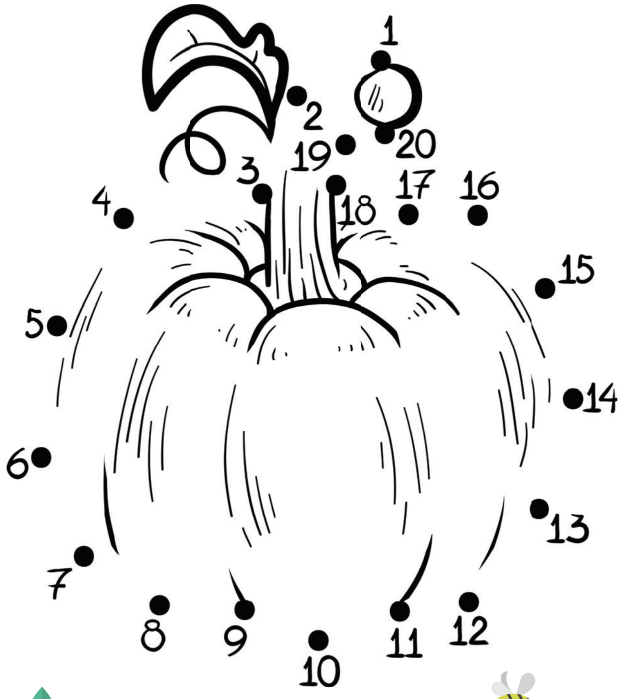

Compre frutas y verduras en temporada

- Manzanas
- Betabel
- Dátiles
- Berenjena
- Espinacas
- Apio
- Tomates
- Pomelo
- Limón
- Naranjas
- Mandarina

Receta de la semana:

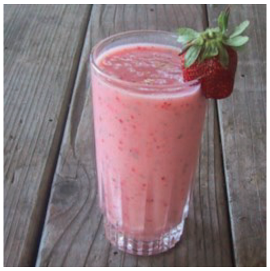

Licuado de jugo de toronja fresco

Ingredientes:

1 1/3 tazas de jugo de toronja roja fresco
8 fresas grandes
2 plátanos medianos, en rebanadas
8 oz yogur de fresa-plátano 2 cucharadas de miel
1 taza hielo picado

Instruccciones: Ponga el jugo de toronja, fresas, plátanos, yogur, miel, y hielo en una licuadora. Cubra, y licúe hasta que no haya grumos.

Para más eventos y recursos, visite nuestro sitio web en https://sites.google.com/asu.edu/sageasu/home?authuser=0

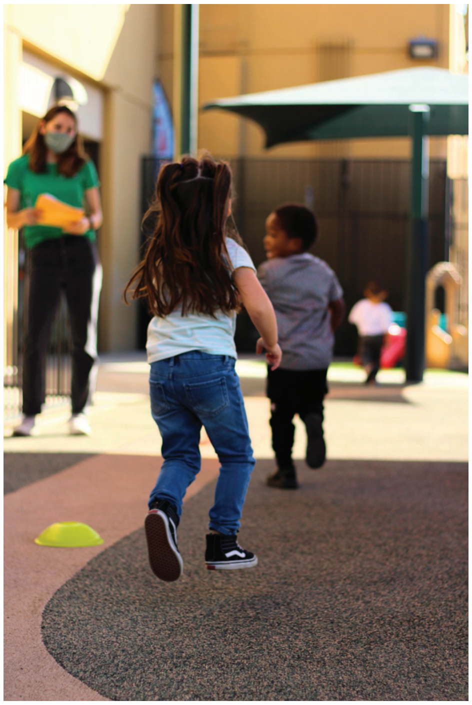

Los niños están hacen actividad física mientras cuidan el jardín. ¡La jardinería puede enseñar habilidades de comunicación y observación!

Usted sabía:

Los supermercados tienen ventas de 3 días. Asegúrese de revisar su anuncio semanal para aprender acerca de las ventas de frutas y verduras del fin de semana.

Para actividad física en familia visite el sitio web abajo para encontrar el parque más cercano a usted. https://www.discovertheforest.org. ¡Seleccione "Locate a forest or park," ponga su código postal y encuentre el parque más cercano!

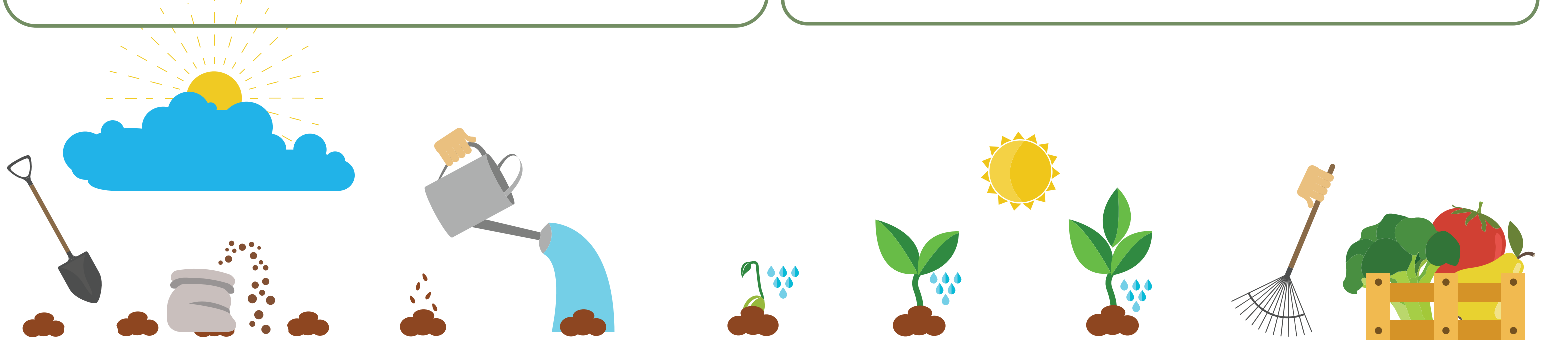

Supplement: Supplementary file 1 [file ijerph-19-04617-s001.zip › ijerph-1609048-supplementary.pdf]
